# Effectiveness analysis of a pharmacist-led intervention for orthopedic perioperative use of antibiotics: a retrospective cohort study

**DOI:** 10.3389/fphar.2024.1365370

**Published:** 2024-11-14

**Authors:** Danwei Wu, Yingxu Li, Jiancun Zhen, Yong Wu, Shuang Ren, Yuan Zhao, Ning Sun, Xuanzi Lin, Liangpeng Lai, Wei Zhang

**Affiliations:** ^1^ Department of Pharmacy, Beijing Jishuitan Hospital, Capital Medical University, Beijing, China; ^2^ Department of Foot and Ankle Surgery, Beijing Jishuitan Hospital, Capital Medical University, Beijing, China

**Keywords:** surgical prophylaxis, antimicrobial stewardship, pharmacist intervention, β-lactam allergy, orthopedic surgery

## Abstract

**Background:**

Following the Chinese guidelines’ recommendation to completely cancel routine cephalosporin skin tests, the choice of cephalosporin as surgical prophylactic medication was affected. This was due to the limited cognition of the predictive value of cephalosporin skin test or the desire to avoid medical disputes. The aim of this retrospective study was to evaluate whether the pharmacist-led perioperative antibiotic prophylaxis model could improve clinicians’ medical behavior in choosing cephalosporin antibiotics for surgical prophylaxis.

**Methods:**

From July 2021 to May 2022, a retrospective analysis was conducted on the selection of surgical preventive medication, skin test, postoperative infection and adverse drug reactions in foot and ankle surgery. The study was divided into three period: the rountine cephalosporin skin test period (Period I: Skin Test), the period when the routine cephalosporin skin test was cancelled but the pharmacist did not intervene (Period II: Cancel Skin Test), and the period when the pharmacist-led perioperative antibiotic prophylaxis was implemented after the cancellation of the cephalosporin skin test (Period III: Pharmacist Intervention).

**Results:**

A total of 1,583 patients were enrolled in this study. There was no significant difference in the utilization rate of cefuroxime between the routine skin test stage and the skin test cancelled stage [74.92% (Period I) vs. 74.54% (Period II), *P* > 0.05]. However, in the pharmacist intervention stage, the usage rate of cefuroxime significantly increased compared to the initial stage when the skin test was cancelled [87.07% (Period III) vs. 74.54% (Period II), *P* < 0.05]. The use of cephalosporins also increased in patients with self-reported beta-lactam allergies between these stages [41.94% (Period III) vs. 3.22% (Period II), *P* < 0.05)]. There was no significant difference in the incidence of postoperative infection and adverse drug reactions among the three periods.

**Conclusion:**

The pharmacist-led perioperative antibiotic prophylaxis model can significantly improve the medical behavior of clinicians in choosing cephalosporin antibiotics as surgical prophylactic medication and optimize the perioperative medication plan.

## 1 Introduction

Surgical site infection (SSI) is responsible for a significant proportion of hospital-acquired infections, ranging from 21% to 36% (Zimlichman et al., 2013; Shelley et al., 2022). Once an SSI occurs, it can lead to persistent infections, prolonged hospital stays, and a substantial increase in medical expenses (Blumenthal et al., 2018). Preventing SSI requires considering various factors such as the type of surgical incision, potential contamination, and the antibiotic sensitivity to specific pathogens. Additionally, it is crucial to ensure that the drug reaches its effective concentration at the surgical site. The Clinical Practice Guidelines for Antimicrobial Prophylaxis in Surgery of the American Society of Health-System Pharmacists (ASHP) and the Chinese guidelines for the clinical application of antibiotic both recommend the use of first- or second-generation cephalosporins as the preferred antibiotics for patients undergoing orthopedic procedures (Bratzler et al., 2013; People’s government of the people’s Republic of China, 2021).

The unpredictable nature of allergic reactions to cephalosporins has raised significant concerns within surgical departments (Zhou et al., 2021). As a result, when a patient is identified as allergic to β-lactam antibiotics, there is often a reluctance to question, re-evaluate, or verify the allergy status to avoid possible adverse reactions. Even patients who self-report as “positive” on skin tests or have a documented “β-lactam allergy history” are typically prescribed alternative antibiotics without further evaluation (Krah et al., 2021). However, recent studies have indicated that anaphylactic shock in patients with anaphylaxis to cephalosporins is extremely rare, occurring from 0.004% to 0.015% (Wilhelm et al., 2022). Furthermore, approximately 80% of patients with a previously positive penicillin skin test and 60% of those with a positive cephalosporin skin test may test negative after 10 or 5 years, respectively (Trubiano et al., 2017; Romano et al., 2014). Therefore, it is essential to accurately evaluate penicillin allergy before ruling out its use or using other β-lactam antibiotics.

The routine use of skin tests to predict cephalosporin allergy remains controversial. Countries such as the United States and South Korea do not recommend regular skin tests before administering cephalosporins (Yoon et al., 2013; Torres et al., 2019). However, in China, requiring routine skin tests before using cephalosporins has been a long-standing practice. This has led to a heavy reliance on the results of skin tests among clinicians, and a positive test often results in patients losing the opportunity to receive β-lactam antibiotics. According to the guidelines, clindamycin can be used as an alternative prophylactic antibiotic for orthopedic surgery. However, studies have shown that clindamycin for surgical prophylaxis in orthopedic surgery may increase the risk of postoperative infection (Linam et al., 2009). Studies have found a higher proportion of patients with β-lactam-allergy who receive clindamycin as a prophylactic antibiotic compared to non-β-lactam-allergic patients who receive first-and second-generation cephalosporins perioperatively (Yian et al., 2020; Coleman et al., 2020). Furthermore, a multicenter prospective cohort study conducted in Canada demonstrated that patients with a documented β-lactam allergy had a three-fold higher risk of adverse events than those without such an allergy (MacFadden et al., 2016).

In 2021, the “Guiding Principles for Skin Testing of β-Lactam Antibacterial Drugs” issued by the China National Health Commission emphasized insufficient evidence to support the clinical predictive value of routine skin tests before administering cephalosporins for allergic reactions. As a result, routine skin tests are not recommended. Consequently, hospitals in China have begun to discontinue regular skin tests for cephalosporins.

Despite the guidelines stating that routine skin tests for cephalosporins are unnecessary, clinicians still express concerns about using cephalosporins without such tests (Bratzler et al., 2013). An analysis of the current status of cephalosporin skin tests in Chinese medical institutions reveals that 42% of hospitals continue to perform skin tests even after the guidelines. However, several studies have shown that involving pharmacists can significantly contribute to the rational use of perioperative antibiotics and promote their appropriate administration during surgical procedures (Zhou et al., 2015; Tang et al., 2018; Shi et al., 2013). Still, more research is needed to demonstrate the impact of pharmacist-led perioperative antibiotic prophylaxis and treatment program on cephalosporin skin tests and proper use of antibiotics.

Following the discontinuation of routine skin tests for cephalosporins at Beijing Jishuitan Hospital, Capital Medical University on 9 November 2021, clinicians may misunderstand the predictive value of cephalosporin skin test, and there are doubts about the selection of cephalosporin without skin test for surgical prophylaxis. Therefore, the pharmacist-led perioperative antibiotic prophylaxis model was implemented in a timely manner, including pharmaceutical consultation, relevant training and so on. The primary objective of this program was to address the problem of incorrect evaluation of patients with β-lactam allergy while promoting the rational use of antibiotics and reducing the incidence of postoperative infections. This retrospective study aimed to assess the impact of the pharmacist-led intervention program on improving clinicians’ medical behavior in choosing cephalosporin antibiotics as surgical prophylaxis.

## 2 Materials and methods

### 2.1 Study design and setting

This retrospective cohort study was conducted at the Department of Foot and Ankle Surgery, Beijing Jishuitan Hospital, Capital Medical University between July 2021 and May 2022, excluding August and November 2021. Because the Department of Foot and Ankle Surgery was relocating to a new district resulting in fewer surgery in August and cefuroxime was temporarily out of stock in November. The study spanned 9 months, consisting of three distinct periods: Period I: Skin Test (from 1 July 2021 to 31 July 2021 and from 1 September 2021 to 31 October 2021):the period with routine cephalosporin skin test; Period II: Cancel skin test (from 1 December 2021 to 28 February 2022), the period when the routine cephalosporin skin test was canceled but the pharmacist did not intervene; and Period III: Pharmacist Intervention (from 1 March 2022 to 31 May 2022), the period when the pharmacist-led perioperative antibiotic prophylaxis model was implemented after the cancellation of the cephalosporin skin test. The study included patients who underwent foot and ankle surgery with a Type I incision. Patients were excluded if they 1) did not have surgical indications after evaluation, 2) opted for conservative treatment instead of surgery, 3) had preoperative infections, such as urinary tract infection or lung infection, and had already received antimicrobial treatment, 4) had a diagnosis of infectious diseases, such as diabetic foot or osteomyelitis, 5) had open grade III fractures characterized by extensive soft tissue damage and crushing, and 6) were transferred to the surgical intensive care unit after operations.

Sample content had to be determined based on the primary objectives of the test. According to the counting data, the correct administration rate of perioperative prophylactic antibiotics was assessed. The correct administration rate of perioperative prophylactic antibiotics in the pharmacist intervention group was 68.75%, and in the control group it was 22.72%; α = 0.05, β = 0.1, a two-sided test was used, and then we used n1 = n2 = 20. A total of 40 patients needed to be included in both groups. Taking into account a 20% loss rate, approximately 48 patients needed to be registered. In this study, each period required a sample size larger than 48.

The primary objective was to analyze and compare the use of prophylactic antibiotics, skin tests and the reduction of the rates of postoperative infections in these three periods. This study was conducted in accordance with the Declaration of Helsinki and was approved by the Institutional Review Board at the Faculty of Ethical Review Approval, Beijing Jishuitan Hospital, Capital Medical University (K2023-371-00).

### 2.2 Pharmacist intervention

We had established a pharmacist-led “clinician-pharmacist-nurse” perioperative management model. Pharmacist interventions were aimed at improving patient care by reducing the misdiagnosis of β-lactam antibiotic allergies and optimizing the prophylactic drug regimen during the perioperative period. Initially, nurses administered a simple questionnaire designed by the clinical pharmacist to conduct a preliminary screening of new patients (Supplementary Table S1). This screening included assessing allergy history and identifying any co-existing medical conditions. Based on the questionnaire results, clinical pharmacists performed additional consultations and reassessments specifically for patients with a history of β-lactam allergy. This reassessment involved evaluating the timing, allergic drugs, specific manifestations, and treatment outcomes associated with the allergy. The allergy history might have been excluded if the symptoms indicated adverse effects, such as gastrointestinal reactions. However, if a drug hypersensitivity reaction was suspected, clinical pharmacists classified the type of reaction according to the Gell-Coombs classification system (Dispenza, 2019). Type Ⅰ hypersensitivity reactions were IgE-mediated allergic reactions, which could include symptoms such as hives, life-threatening allergic shock, bronchial asthma, and laryngeal edema. These reactions typically occurred within 1 h after drug administration. Delayed-type hypersensitivity reactions, including type II, III, and IV, usually manifested 1 h or more after drug administration. It was important to exclude cases that could be mistaken for “allergies,” such as patients who reported positive skin tests for penicillin/cephalosporin in childhood but could tolerate oral cephalosporin or amoxicillin. Finally, based on the re-evaluation outcomes, clinical pharmacists communicated with the clinicians to determine the optimal antibiotics for surgical prophylaxis.

**FIGURE 1:**
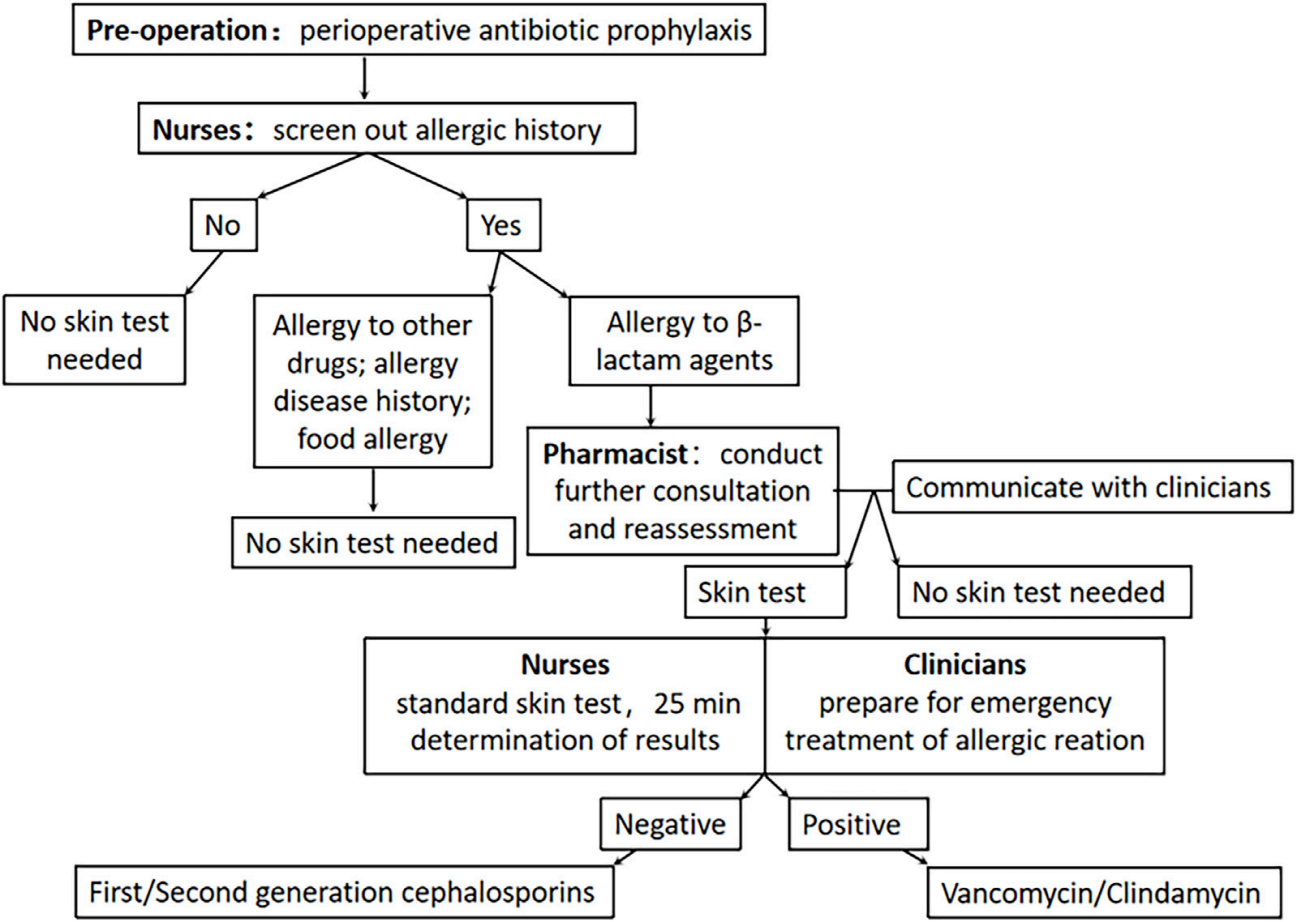
Pharmacist-led “clinician-pharmacist-nurse” perioperative management model.

Clinical pharmacists actively participated in daily ward rounds to quickly identify issues and collaborate with clinicians to formulate perioperative treatment plans. Conducting pharmacy ward rounds and pharmaceutical consultations for patients of special concern. As part of the intervention, clinical pharmacists provided periodic training for clinicians and nurses on antibiotic prophylaxis and topics related to skin testing. These training sessions addressed common issues encountered in daily medical care, such as distinguishing between patients who were truly allergic to β-lactams and those who experienced adverse reactions due to inappropriate medication or other factors. The detailed The detailed pharmacist-led “clinician-pharmacist-nurse” perioperative management model intervention process flow is shown in Figure 1.

### 2.3 Data collection

The following demographic and clinical data were collected from the medical record: sex, age, height, weight, length of hospital stay, and existing medical conditions or comorbidities. Surgical records were reviewed to collect data on the type of surgery performed (arthroscopic or non-arthroscopic procedures). Additional surgical details, including the operation time and intraoperative blood loss were also recorded.

Data on food allergies, drug allergies, and allergic diseases were collected to assess patients’ history of allergies. Information on skin tests, including the number of tests conducted, was documented. The usage rate of perioperative prophylactic antibiotics was recorded. The occurrence of infections during the perioperative period was also noted. Adverse reactions were determined based on Naranjo’s assessment (Belhekar et al., 2014), a standardized method for assessing adverse drug reactions.

### 2.4 Statistical analysis

Data were analyzed using SPSS software (version 26.0; SPSS, Inc, Chicago, IL). Testing the normality of continuous variables was performed using the Kolmogorov-Smirnov test. If the distribution was consistent with normality, it was expressed as the mean and standard deviation. If it was not, the distribution was represented by the median and quartiles, while the categorical variables were presented as frequencies and percentages. The Pearson chi-square test was used to analyze categorical data, while variance analysis was used to compare continuous variables (age, BMI, length of hospital stay, etc.). Statistical significance was assessed separately for each test. A *p*-value of less than 0.05 was considered statistically significant.

## 3 Results

### 3.1 Characteristics of the study population

**FIGURE 2:**
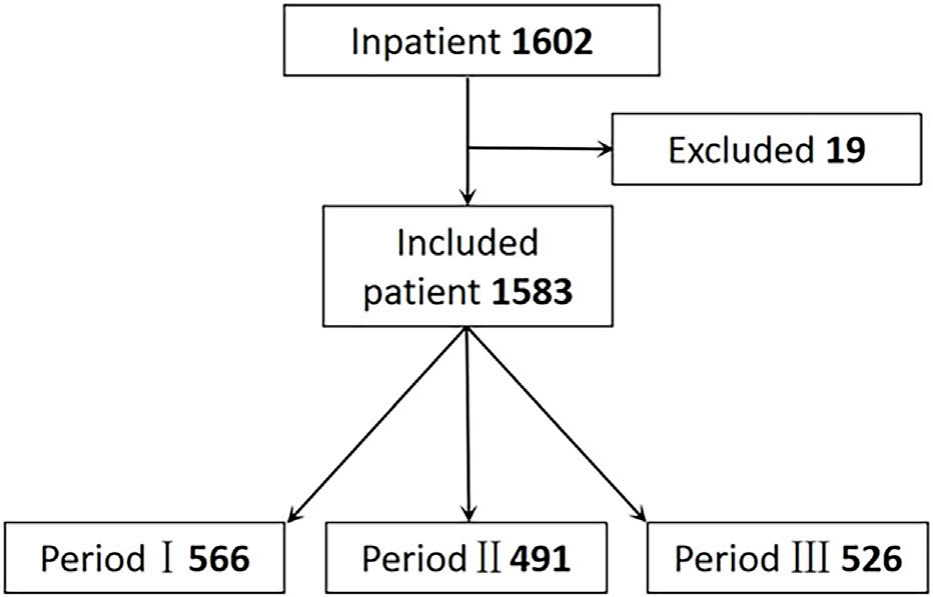
Flow of relection criteria. Period I: Skin test; Period II: Cancel Skin test; Period Ⅲ: Pharmacist intervention.

During the three periods, 1,602 patients were admitted, and 1,583 were included in the analysis. 19 cases were excluded due to conservative treatment, preoperative infection and so on. Among these were 566 patients in Period I, 491 in Period II, and 526 in Period III, as Figure 2 shows.

There were no statistical differences in sex, age, length of hospital stay, BMI, and comorbidity between the three periods, as indicated in Table 1.

**TABLE 1:** Demographic and clinical data of the three periods.

Demographic and clinical data	Period I (n = 566)	Period II (n = 491)	Period III (n = 526)	*P*
Age, years	43.23 ± 17.36	43.29 ± 15.72	44.01 ± 15.42	0.306
Female (n, %)	260 (45.94)	275 (56.01)	279 (53.04)	0.348
Hospital stay, days	6.65 ± 2.09	6.57 ± 1.79	6.70 ± 2.08	0.706
BMI (kg/m^2^)	24.31 ± 4.52	25.47 ± 7.44	25.14 ± 4.21	0.129
Comorbidity (n, %)				
Hypertension	65 (11.48)	78 (15.89)	104 (19.77)	0.281
Diabetes	28 (4.95)	29 (5.91)	39 (7.37)	0.739
Cardiovascular disease	14 (2.47)	13 (2.65)	23 (4.35)	0.723
Hyperlipidemia	11 (1.94)	11 (2.24)	23 (4.35)	0.742
Gout	10 (1.77)	13 (2.65)	13 (2.46)	0.314
Rheumaticimmune diseases	7 (1.24)	11 (2.24)	13 (2.46)	0.203
Respiratory diseases	6 (1.06)	8 (1.63)	5 (0.95)	0.273
Tumor	3 (0.53)	3 (0.61)	4 (0.76)	0.786
Hepatobiliary system diseases	3 (0.53)	3 (0.61)	4 (0.76)	0.786
Digestive system diseases	3 (0.53)	4 (0.81)	9 (1.70)	0.706
Thyroid disease	6 (1.06)	7 (1.43)	11 (2.10)	0.512
Urinary system diseases	2 (0.35)	1 (0.20)	11 (2.10)	0.685
Nervous system diseases	5 (0.88)	7 (1.43)	7 (1.32)	0.526
Others	5 (0.88)	3 (0.61)	8 (1.51)	0.699

Period I: Skin test; Period II: Cancel skin test; Period III: Pharmacist intervention.

There were no significant differences in the operation time and intraoperative blood loss between the three periods (*P* > 0.05). However, a significant increase in the number of arthroscopic surgeries was observed after discontinuing the skin test practice compared to the period before the discontinuation of the skin test (*P* < 0.01), since the department of foot and ankle surgery began to use new surgical techniques, the number of arthroscopic surgeries increased in Period II and Period III, as indicated in Table 2.

**TABLE 2:** Surgical information.

Surgical information	Period I (n = 566)	Period II (n = 491)	Period III (n = 526)	*P*
Arthroscopic surgery (n, %)	92 (16.25)	122 (24.84)	123 (23.38)	<0.01
Operation time (min)	102.91 ± 3.27	99.77 ± 1.57	97.14 ± 1.04	0.154
Bleeding amount (mL)	24.66 ± 2.77	18.78 ± 1.56	19.94 ± 1.04	0.051

Period I: Skin test; Period II: Cancel skin test; Period III: Pharmacist intervention.

### 3.2 Allergy history and allergic diseases

The patient’s allergy history and allergic diseases were analyzed across the three periods, Supplementary Table S2 shows patients with a history of antibiotic allergy, while Supplementary Table S3 shows patients who had allergic diseases. The allergy to β-lactam agents was the most prevalent among the three periods, representing 3.53%, 12.63%, and 11.79% of the total patient population in Periods I, II, and III, respectively. Additionally, the self-reported β-lactam agents allergy rate was significantly lower in Period I compared to Periods II and III (*P* < 0.01). Among patients with allergic diseases, allergic rhinitis was the most common, accounting for 0.71%, 0.81%, and 0.57% in Periods I, II, and III, respectively.

### 3.3 Antibiotics for surgical prophylaxis

**FIGURE 3:**
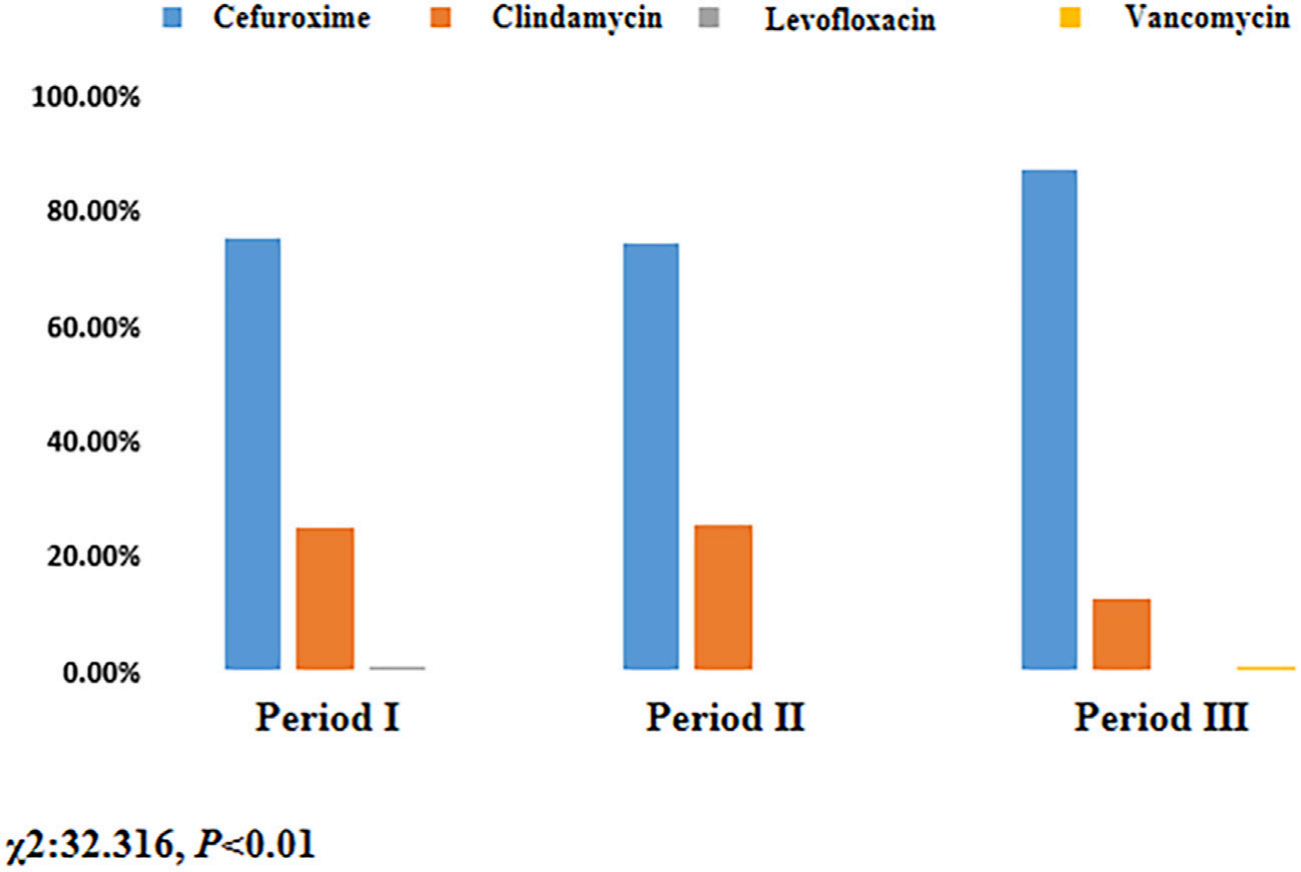
Proportion of patients using different antibiotic for surgical prophylaxis. Period I: Skin test; Period II: Cancel skin test; Period Ⅲ: Pharmacist intervention.

**FIGURE 4:**
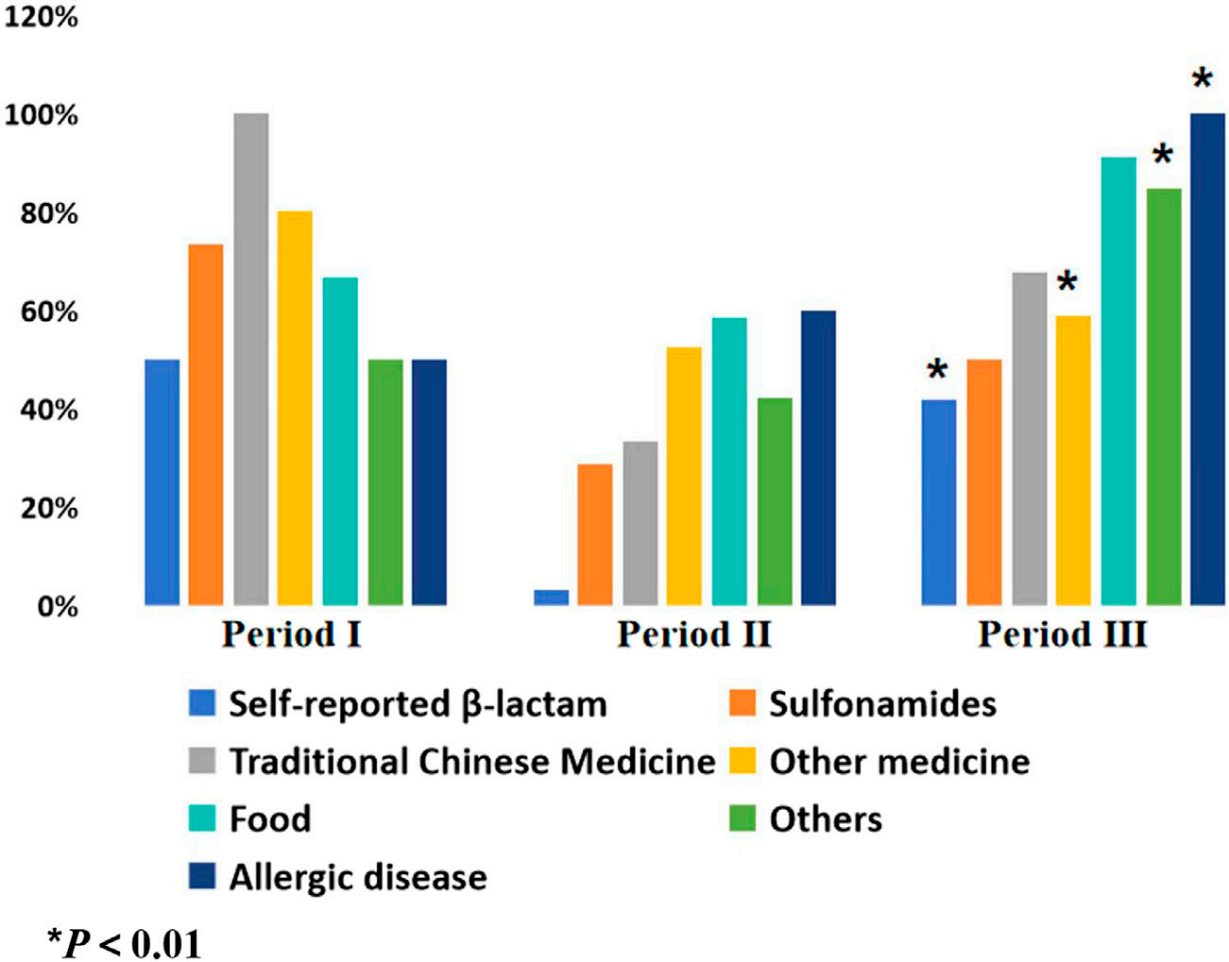
Proportion of patients with different allergy history using cefuroxime for surgical prophylaxis. Period I: Skin test; Period II: Cancel skin test; Period Ⅲ: Pharmacist intervention.

There was no significant increase in the use of cefuroxime in Period II compared to Period I (74.54% vs. 74.92%, *P* > 0.05), as indicated in Table 3. However, the rate of cefuroxime used significantly increased in Period III with pharmacist interventions compared to Period II (87.07% vs. 74.54%, *P* < 0.05). In contrast, the clindamycin use rate significantly decreased due to pharmacist intervention in Period III compared to Period II (12.74% vs. 25.46%, *P* < 0.05), as indicated in Figure 3. The cefuroxime use rate as surgical prophylaxis in patients who claimed β-lactam allergy was significantly increased in phase III compared to phase II (41.94% vs. 3.22%, *P* < 0.01), as shown in Table 4. This outcome demonstrates that pharmacists’ intervention significantly improved the rate of cephalosporin use. Additionally, among patients with other types of allergies (such as pollen, animal hair, dust mites, etc.), the rate of using cephalosporin after intervention also increased (Period I: 50%, Period II: 42.11%, Period III: 84.62%, *P* < 0.01). Figure 4 provides a clear visual representation of the proportion of patients with different types of allergies who used cefuroxime as surgical prophylaxis.

**TABLE 3:** Proportion of patients using different antibiotic for surgical prophylaxis.

Antibiotic (n, %)	Period I (n = 566)	Period II (n = 491)	Period III (n = 526)
Cefuroxime^a^	424 (74.92)	366 (74.54)	458 (87.07)
Clindamycin	140 (24.73)	125 (25.46)	67 (12.74)
Levofloxacin	2 (0.35)	0 (0.00)	0 (0.00)
Vancomycin	0 (0.00)	0 (0.00)	1 (0.19)

^a^ χ2:32.316, *P* < 0.01; Period I: Skin test; Period II: Cancel skin test; Period III: Pharmacist intervention.

**TABLE 4:** Proportion of patients with allergy history using cefuroxime for surgical prophylaxis.

Type of allergy (n, %)	Period I	Period II	Period III	χ2	*P*
Self-reported β-lactam	10 (50.00)	2 (3.22)	26 (41.94)	30.579	<0.01
Sulfonamides	11 (73.33)	4 (28.57)	9 (50.00)	5.819	0.054
Traditional Chinese medicine	1 (100.00)	1 (33.33)	2 (66.66)	1.556	0.459
Other medicine	8 (80.00)	11 (52.38)	10 (58.82)	18.358	<0.01
Food	4 (66.66)	7 (58.33)	21 (91.30)	5.535	0.063
Others	7 (50.00)	8 (42.11)	22 (84.62)	10.570	<0.01
Allergic disease	4 (50.00)	6 (60.00)	8 (100.00)	66.667	<0.01

Period I: Skin test; Period II: Cancel skin test; Period III: Pharmacist intervention.

### 3.4 Skin test

After discontinuing the routine skin test policy, the proportion of patients undergoing skin tests decreased significantly from 100% to 2.85%. Subsequently, following pharmacist intervention, the proportion increased to 6.08%, indicating a statistically significant difference (*P* < 0.01). Supplementary Table S4 summarizes the proportion of patients undergoing skin tests.

The routine skin test policy also resulted in unnecessary expenses, including antibiotic and operation costs.

### 3.5 Adverse reactions and infection

According to Knott’s assessment, we analyzed the adverse reactions that occurred following antibiotic infusion. In Period Ⅱ, three cases of adverse drug reactions were reported after clindamycin infusion: one case of nausea and vomiting, one of heart discomfort and one of rash. In Period Ⅲ, one case of adverse reaction occurred after cefuroxime infusion, the patient reported heart discomfort and rash.

Furthermore, we recorded the incidence of infection within 30 days after surgery, focusing on the main types, including superficial incision, deep incision, and organ/space infection. As a surgical prophylactic medication, cefuroxime was associated with two cases of skin and soft tissue infections reported in Period I, and one case of osteomyelitis reported in Period III. Clindamycin, used similarly as a surgical prophylactic, resulted in one case of osteomyelitis reported in Period II, and during Period III, one additional case of osteomyelitis and one case of skin and soft tissue infection were reported. As shown in Supplementary Tables S5, S6, no significant differences were observed in the incidence of adverse reactions or infections between the different periods (*P* > 0.05).

## 4 Discussion

In the clinical setting, the presence of a “β-lactam allergy” label had a detrimental impact on various aspects of patient care, including antibiotic selection, relevant clinical outcomes (such as hospital stay and surgical complications), and economic costs (Krah et al., 2021; Plessis et al., 2019; Blumenthal et al., 2019). The routine skin test for cephalosporins significantly contributed to misidentifying patients as allergic. However, insufficient evidence supported the clinical predictive value of this skin test for allergic reactions (Vaisman et al., 2017; Macy, 2014). Consequently, many patients were misidentified as having “β-lactam allergies,” leading to missed opportunities for appropriate antibiotic use.

Despite the guideline issued in China in 2021 to discontinue routine skin tests for cephalosporins, clinicians still had concerns and tended to increase clindamycin as an alternative. Several studies had shown that although approximately 8%–20% of patients’ self-reported β-lactam allergies, more than 95% could safely tolerate penicillin/cephalosporin (Coleman et al., 2020; Osei and Boyer, 2012). The reasons for this discrepancy may have included patients who inaccurately expressed their symptoms, inappropriate differentiation between allergies and adverse reactions, and healthcare staff having misconceptions about allergy histories (Osei and Boyer, 2012; Li et al., 2014; Blumenthal et al., 2014; Shah et al., 2016). Therefore, conducting more consultations and evaluations of patients was crucial to accurately assess their allergic status. In our study, clinical pharmacists played a significant role in evaluating whether a patient had a true allergy or an adverse reaction and promoted the rational use of antibiotics by periodically disseminating relevant knowledge to clinicians and nurses. This collaborative effort helped ensure appropriate antibiotic selection and usage in the clinical setting.

This study showed through the pharmacist-led perioperative antibiotic prophylaxis model, the use of cephalosporin antibiotics as surgical prophylaxis was standardized, especially in patients with self-reported β-lactam allergy, thereby eliminating the need for clinicians to misunderstand routine cephalosporin skin testing and reducing unnecessary waste of resources and medical expenses.

The participation of clinical pharmacists in chronic disease management, such as diabetes, hemodialysis, and hypertension, had been well-established. This participation showed significant improvements in clinical indicators and yielded positive economic benefits, establishing clinical pharmacists as vital members of the medical team (Blumenthal et al., 2014; Shah et al., 2016; Bald et al., 2022). However, there were limited data on the effective intervention of clinical pharmacists in the perioperative period, particularly studies that included clinical pharmacists as part of the management team. Clinical pharmacists were crucial in addressing issues related to the rational use of perioperative antibiotics (Martin et al., 2018). Studies had shown that clinical pharmacist interventions promoted the rational use of prophylactic antibiotics and resulted in favorable economic outcomes (Omboni et al., 2019; Zhou et al., 2023). Furthermore, a prospective cohort study demonstrated that clinical pharmacist interventions improved the appropriate use of antibiotics and reduced hospital stay duration and associated costs (Butt et al., 2019). In our study, clinical pharmacists played a significant role in evaluating whether a patient had a true allergy or an adverse reaction and promoted the rational use of antibiotics by periodically disseminating relevant knowledge to clinicians and nurses. This collaborative effort helped ensure appropriate antibiotic selection and usage in the clinical setting. It included questioning of medical history and allergy history before medication, close observation of the patient’s reaction after medication, and improvement of emergency rescue measures and other multi-faceted monitoring. On the one hand, from the patient’s perspective, pharmacist-led perioperative antibiotic prophylaxis model could save drug costs, consumables, nurse labor costs, etc. On the other hand, from the perspective of hospital operation, the operation time and observation time of a skin test were about 25 min, which could save nurses’ time.

As for postoperative infection, there was no statistical difference between the three period. The reason may have been that foot and ankle surgery had certain particularities. The elective surgery of foot and ankle surgery was compared with that of orthopedics and spine surgery in terms of operation time and blood loss, and it was relatively small. According to literature reports, the incidence of infection 30 days after a type of incision in foot and ankle surgery was generally low at 0.48%–4.3% (Paul et al., 2015). Therefore, there was no difference in the incidence of infection 30 days after surgery in this study. Besides there was no statistical difference between the three period on adverse reactions. According to literature reports, it was difficult to define allergic reactions to cephalosporins and other adverse reactions, and their incidence rate accounted for 1%–3% of the total population (Jean et al., 2006), in this study the incidence of adverse reactions in the three period of the study was low. The reason may have been that this study was a retrospective study and there may have been data loss, so we did not obtain ideal results in this part. Even though there was no statistical difference in the above results, the author believed that the intervention of clinical pharmacists had increased the use rate of cephalosporins in perioperative prophylaxis. This result could prove the effectiveness of clinical pharmacists’ intervention and promoted the use of cephalosporins as selection of perioperative surgical prophylaxis. At this period, there had been a large number of prospective studies showing that cephalosporins were superior to clindamycin in terms of safety and effectiveness, and their status as a preventive drug for incisions in orthopedic surgery was unquestionable.

Our study aimed to establish a comprehensive pharmacist-led perioperative antibiotic prophylaxis model, which efficiently screened patients with underlying conditions and an allergy history while promoting the rational use of prophylactic antibiotics. The results demonstrated that not only does this model produce economic benefits and reduce labor costs, but it also improved the appropriate utilization of antibiotics. Based on these findings, the pharmacist-led perioperative antibiotic prophylaxis model we established had great potential for clinical application. The clinical pharmacists’ interventions in this model extended beyond promoting the rational use of antibiotics and could contribute to advancing surgical perioperative care. Clinical pharmacists could further enhance patient care and optimize outcomes in surgical settings by providing guidance and assistance at the pharmaceutical care level through a multidisciplinary diagnosis and treatment approach.

This study had some limitations. First, it was a retrospective cohort study, which might not have provided as strong evidence as randomized controlled trials. Therefore, the findings of our study should be interpreted with caution. Second, we did not observe significant differences in postoperative infections related to type I incisions or adverse reactions caused by antibiotics. It was essential to note that the low infection rate after the type I incision and the small sample size could have influenced these results. A larger sample size or a different study design is needed to provide more conclusive evidence of these outcomes.

## 5 Conclusion

The findings of this study demonstrated the positive impact of pharmacist-led perioperative antibiotic prophylaxis model in promoting the rational use of prophylactic antibiotics during the perioperative period. The model significantly increased the use of second-generation cephalosporins, particularly in patients with a history of drug allergies, including β-lactam allergy. By leveraging pharmacists’ expertise and fostering collaborative decision-making among healthcare professionals, the model contributed to better patient outcomes and saved medical costs.

## Data Availability

The raw data supporting the conclusions of this article will be made available by the authors, without undue reservation.
