# Supplementary material for: Effectiveness analysis of a pharmacist-led intervention for orthopedic perioperative use of antibiotics: a retrospective cohort study
Source: Front Pharmacol. 2024 Nov 14;15:1365370. doi: 10.3389/fphar.2024.1365370 (PMC11604034; doi:10.3389/fphar.2024.1365370)
Supplement: Supplementary file 1 [file Presentation1.pdf]

frontiers

Supplementary Material

Effectiveness analysis of a pharmacist-led intervention for orthopedic perioperative use of antibiotics: A retrospective cohort study

Danwei Wu[1]†, Yingxu Li[1]†, Jiancun Zhen[1], Yong Wu[2], Shuang Ren[1], Yuan Zhao[2], Ning Sun[2], Xuanzi Lin[1], Liangpeng Lai[2]* and Wei Zhang[1]*
1 Department of Pharmacy, Beijing Jishuitan Hospital, Capital Medical University, Beijing 100035, China
2 Department of Foot and Ankle Surgery, Beijing Jishuitan Hospital, Capital Medical University, Beijing 100035, China
*** Correspondence:**
Wei Zhang :jstyjkky@126.com； Liangpeng Lai:downye@163.com

†These authors contributed equally to this work and share first authorship

Supplementary Table

Table S1. Patient questionnaire

**Number:**	**Name:**	**Patient's signature:**	**Nurse's signature:**
1. Do you have any allergies to drugs, food, or others?			Yes/No
2. Do you have a history of cephalosporin allergy?			Yes/No
3. Do you have a history of penicillin allergy?			Yes/No
4. What are the symptoms of allergies? Is there an anaphylactic shock?			Yes/No

Table S2. Patient's allergy history

**Type of allergy (n, %)**		**Period I (n=566)**	**Period II (n=491)**	**Period III (n=526)**	***P***
Drug	β-lactam agents	20(3.53)	62(12.63)	62(11.79)	<0.01
	Sulfonamides	15(2.65)	14(2.85)	18(3.42)	0.741
	Traditional Chinese Medicine	1(0.18)	3(0.61)	3(0.57)	0.492
	Other medicine	10(1.77)	21(4.28)	17(3.23)	0.589
Food	Sea food	3(0.53)	2(0.41)	9(1.71)	0.364

	Fruits	0(0.00)	5(1.02)	7(1.33)	0.604
	Others	3(0.53)	5(1.02)	7(1.33)	0.165
Others	Pollen	4(0.71)	6(1.22)	11(2.09)	0.162
	Alcohol	2(0.35)	6(1.22)	2(0.38)	0.685
	Animal hair	3(0.53)	2(0.41)	0(0.00)	0.207
	Others	5(0.88)	5(1.02)	13(2.47)	0.056

Period I: Skin test; Period II: Cancel skin test; Period III: Pharmacist intervention

Table S3. Patients with allergic diseases

Type (n, %)	Period I (n=566)	Period II (n=491)	Period III(n=526)	*P*
Allergic rhinitis	4(0.71)	4(0.81)	3(0.57)	0.864
Asthma	3(0.53)	2(0.41)	4(0.76)	0.405
Hives	1(0.18)	2(0.41)	1(0.19)	0.876
Eczema & allergic dermatitis	0(0.00)	2(0.41)	0(0.00)	0.238

Period I: Skin test; Period II: Cancel skin test; Period III: Pharmacist intervention

Table S4. Proportion of patients undergoing skin testing

Proportion (n, %)	Period I (n=566)	Period II (n=491)	Period III (n=526)	*P*
Skin test of patient	566(100%)	14(2.85%)	32(6.08%)	<0.01

Period I: Skin test; Period II: Cancel skin test; Period III: Pharmacist intervention

Table S5. Information of patients' adverse reactions after using perioperative antibiotics

Antibiotic (n, %)	Period I	Period II	Period III	*P*
Cefuroxime	0(0.00)	0(0.00)	1(0.22)	0.386
Clindamycin	0(0.00)	3(2.4)	0(0.00)	

Period I: Skin test; Period II: Cancel skin test; Period III: Pharmacist intervention

Table S6. Proportion of patients with postoperative infection after using perioperative antibiotics

**Antibiotic (n, %)**	**Period I**	**Period II**	**Period III**	***P***
Cefuroxime	2(0.47)	0(0.00)	1(0.22)	0.199
Clindamycin	0(0.00)	1(0.80)	2(2.99)	

Period I: Skin test; Period II: Cancel skin test; Period III: Pharmacist intervention
